# Use of Glass Ionomer Cement for Incudostapedial Rebridging Ossiculoplasty

**DOI:** 10.22038/ijorl.2020.46375.2518

**Published:** 2021-03

**Authors:** Ankur Mohan, Sanjeev Bhagat, Dimple Sahni, Gurkiran Kaur

**Affiliations:** 1 *Department Of * *Otolaryngology* *, Head and Neck Surgery, Government Medical College, Patiala, India.*

**Keywords:** Chronic otitis media, Glass ionomer cement, Ossiculoplasty, Tympanoplasty

## Abstract

**Introduction:**

The ossicles of the middle ear are affected by the erosive effect of pathology in chronic otitis media. Ossicular reparation can be done via autologous incus or with alloplastic materials. Glass ionomer cement (GIC) is simple to use and saves considerable operative time and expenses especially in developing countries where costly ossicular prosthesis are not affordable for the majority of the patients.

**Materials and Methods:**

Twenty-five chronic otitis media patients who underwent surgery were included in this study. The reconstruction material used in this study was glass ionomer cement. All patients had erosion of the long process of incus and a normal stapes.

**Results:**

Pure tone average in pre-operative and post-operative period of study patients were 50.09 & 29.92 dB respectively (P=0.01) and the air-bone gap was 24.85 dB preoperatively and 14.05 dB postoperatively. The closure of the air-bone gap was statistically significant (P= 0.01).

**Conclusion:**

The study showed that the use of GIC ossiculoplasty is an efficient method for the reparation of the long process of the incus. The results are encouraging and indicate that it is worthwhile to conduct more trials using this method.

## Introduction

Chronic otitis media (COM) is a long-standing infection (>12 weeks) of a part or whole of the middle ear cleft characterized by ear discharge and a persistent perforation. It affects both sexes and every age group. 

Ossicular erosion, a frequent complication of COM, may lead to malfunctioning of middle ear mechanics, which results in considerable hearing loss (1). The attrition of ossicles can be observed in tubotympanic as well as atticoantral types of COM (2). The long process of incus is most often involved, followed by stapes crura, body of incus, and manubrium. The erosion of the long process of incus and stapes structure is due to their delicate structure and location. Erosion leads to incudo-stapedial gap causing a conductive hearing loss (3). Several techniques are suggested for ossiculoplasty since the 1950s (4,5). Every technique and prosthesis has some pros and cons and no single ossiculoplasty procedure has received widespread acceptance (6).

Glass ionomer cement (GIC) was developed by the chemist's Alan Wilson and Brian Kent in the 1970s (7-9). Initially, this material was used in orthodontics; however, later, it has been used for skull defect repair, mastoid obliteration, stabilization of various implants, as well as for ossiculoplasty, tegmen, and external auditory canal wall reconstruction (10).

GIC is easy to use and find material (11,12). It is readily available at dental care products shop at an economical price. The GIC is composed of glass powder, polycarboxylic acid, and pigments, as well as a liquid consisting of water, tartaric acid, and conservation agents. After combining, the materials form bone-like consistency in 5–10 minutes.

This study aimed to evaluate the results and outcomes of Glass ionomer cement as a candidate for ossiculoplasty due to defects of the long process of the incus in safe COM.

## Materials and Methods

This prospective and observational study was conducted in our institution on 25 patients with COM who underwent ossicular reconstruction with GIC.

Inclusion criteria include patients with mucosal COM, having conductive hearing loss, and diagnosed with incudo-stapedial discontinuity (intraoperative), and age >10 years. Exclusion criteria were patients with unsafe COM, having otosclerosis with stapes fixation, cholesteatoma, tympanosclerosis, eustachian tube dysfunction, and active otitis media.

Patients satisfying inclusion criteria were admitted one day before surgery and history taking, complete ENT examination was done. Pure tone audiometry and X-ray lateral view mastoids were done. Written informed consent was obtained from all the patients preoperatively. 

Tympanoplasty with ossiculoplasty was performed under general anesthesia in all the patients. The procedure was carried out using conchal cartilage or temporalis fascia, harvested intraoperatively from the patient.

**Fig 1:**
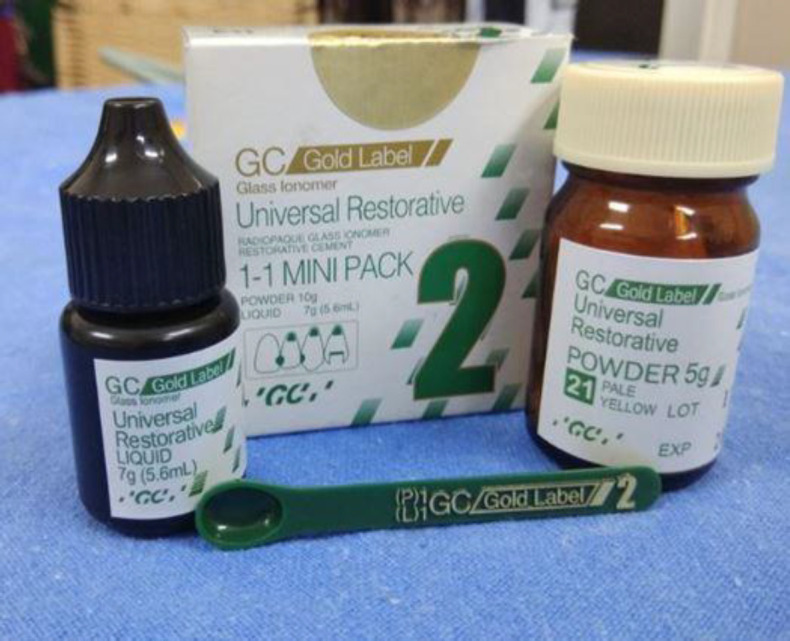
Glass Ionomer Cement

The post-auricular approach was used. After harvesting the temporalis fascia/conchal cartilage graft, the middle ear was examined for any pathology. During the surgical procedure, the condition of the ossicular chain was noted, and any incudo-stapedial gap was reconstructed using GIC. GIC comprises two containers of powder and liquid each (Fig.1). 

One drop of liquid and one scoop of powder (measured with scoop provided along with the product) were mixed on a glass slide, till it becomes uniformly consistent. This mixture hardens in about two minutes. It is required that we perform ossiculoplasty in these two minutes before the mixture hardens. A straight pick was used for the gradual application of cement over the ossicular gap between incus and stapes (^Fig's.2^,3). Generally, one to two applications were sufficient to make an ossicular bridge between incus and stapes. 3-5 minutes was given for the setting of the cement. This bridge now formed strengthens and form a continuity between ossicles (Fig.4). Ossicular mobility was checked and was found to be restored. TM graft placement and incision closure are done in a standard manner. 

**Fig 2:**
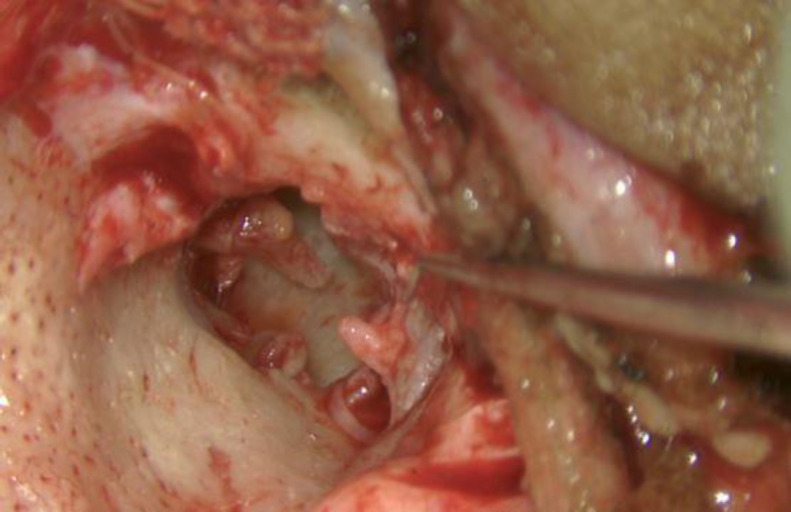
Intraoperative Photograph Showing Incudos- tapedial Discontinuity

**Fig 3:**
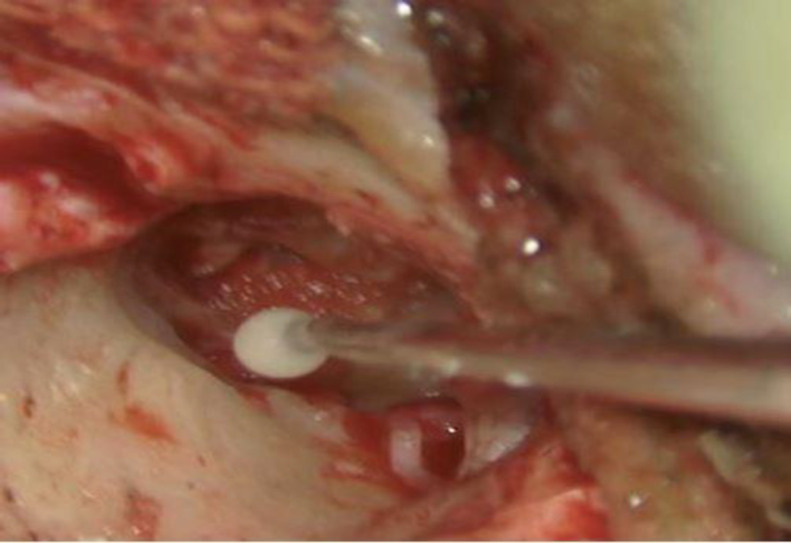
Intraoperative Glass Ionomer Cement Application

**Fig 4:**
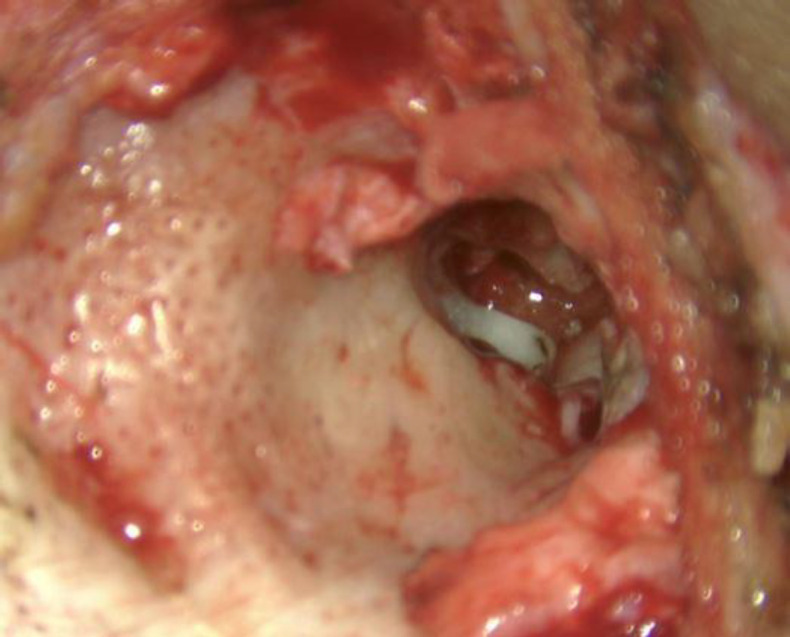
Intraoperative Photograph Showing Incudos- tapedial Joint Restoration after Glass Ionomer Cement Application

Antiseptic dressing was done. Patients were observed for graft uptake and any complications at 4th, 6th, and 12th weeks of operation. If the graft uptake was present at the 12th week, the patient’s pure tone audiometry was done to assess the hearing. PTA was repeated at the 16^th^week after surgery and was compared with that of the preoperative audiogram. All the audiograms were done by the same audiologist in a regularly calibrated audiometer in the department. The air-bone gap (ABG) was calculated for each patient. Improvement in the hearing was considered if ABG <20 dB after 4 months of surgery.

## Results

Twenty-five patients satisfying the inclusion criteria were considered for our study. The age of patients ranged between 13 and 53 years. The mean age was 28.04 (standard deviation [SD 10.39]). Most of the patients comprised of the age group of 21–30 years. Of the 25 patients,6 were female patients (24%) and 19 were male patients (76%).In this study, the most common complaint was ear discharge in 16 cases (64%) followed by ear discharge with decreased hearing in 9 (36%) cases. The medium central perforation was present in 16 (64%) patients whereas large central perforation was present only in 9 (36%).

Student’s paired *t*‑test was applied to the collected data to analyze the gain in hearing at various prespecified intervals with confidence interval set at 99.99% with P< 0.001 considered significant.

Graft uptake was observed in 21 (84%) patients. Pure-tone average (PTA) was calculated using 0.5, 1, 2, and 3 kHz (according to AAO-HNS criteria) AC thresholds. Preoperative PTA AC was 50.09±9.32 dB. Mean postoperative hearing improvement at 3 months was 38.89±10.61 dB and that at 4 months was 29.92±10.08 dB, which was highly significant.

The mean preoperative PTA AB gap was 24.85±4.95dB. The mean postoperative hearing improvement at 3 months in the PTA AB gap was 15.63±7.31dB and that at 4 months was 14.05±7.11dB. Postoperative AB gap closure (PTA AB gap < 20 dB at 16 weeks) was observed in 19 of the 25 patients, which was highly significant.

## Discussion

Hearing impairment is the most common sequel of COM occurring because of TM perforation and possibly erosion of ossicular chain, resulting in conductive hearing loss ranging from 20 to 60 dB (13). Incus is considered to be the most commonly eroded ossicle. To compensate for the impedance matching deficit caused by ossicular disruption, several surgical modalities such as autologous cartilage or sculpted ossicle, allograft material usage, hydroxyapatite cement, and GIC have been used. The results of ossiculoplasty even by experienced otologists with techniques with proven success rates, hearing loss persists in some patients (14). The reparation of the ossicular gap rather than replacement could preserve ossicular chain efficiency and also eliminate the risk of extrusion of the prosthesis. In the following discussion, we tried to evaluate the use of GIC as a potential material for ossicular chain reconstruction and also to review if there were any side effects of its use in the middle ear. As per the American Academy of Otolaryngology hearing evaluation guidelines, thresholds at 500, 1000, 2000, and 3000 hertz were used to calculate the PTA. The ABG was calculated for each patient and the results were tabulated. Improvement in the hearing was considered if ABG < 20 dB after 4 months of surgery. This was observed in 19 (76%) of the 25 patients. Mean preoperative PTA AC was 50.09±9.32, which improved to 29.92±10.08 at 16 weeks. 

The mean preoperative PTA AB gap was 24.85±4.95, which was reduced to 14.05±7.11 at 16 weeks. This was highly significant. Graft uptake was observed in 21 (84%) of the 25 patients. 

In the literature, the reconstruction of incudo-stapedial defects with various bone cement materials has been reported in several patients (Table.1). Our results with GIC are identical to other studies reporting this technique as successful.

**Table 1:** Results of various studies on the use of GIC for ossiculoplasty

**Sr. no.**	**Authors**	**Year**	**No. of patients**	**Follow-up period**	**ABG< 20%**	***P***
1.	Feghali et al. (15)	1998	5	3–6 months	80%	<0.01
2.	Ozer et al. (12)	2002	15	1 year	60%	<0.01
3.	Babu and Seidman (17)	2004	18	1 year	94.4%	<0.01
4.	Bayazit et al. (18)	2005	42	9–40 months	78.6%	<0.01
5.	Baglam et al. (19)	2009	136	1 year	81.6%	<0.01
6.	Celik et al. (20)	2009	29	1 year 11 months	94%	<0.001
7.	Dere et al. (21)	2011	23	1 year	–	<0.01
8.	Somers et al. (22)	2011	10	6–12 months	80%	<0.01
9.	Kalkioglu et al. (23)	2012	42	6 months	76%	<0.001
10.	Celenk et al. (16)	2013	50	1 year	63.2 %	<0.0186
11.	Yogeesha et al. (24)	2018	30	3 months	66.67%	<0.01
12.	Present study	2018	25	4 months	76%	<0.01

Hypothetically, after a successful graft uptake and in normal middle ear volume, one can expect improvement in hearing on PTA. In routine practice, this is not seen after every tympanoplasty. If the volume of the middle ear after tympanoplasty is normal and neotympanum makes appropriate contact with the handle of malleus, ABG in the normal range is expected, still, in some cases, ABG is narrower than that suggestive of ossicular discontinuity. It is difficult to explain ABG in the range of 10-20 dB. The cause of failure in hearing improvement may be fixed ossicles or some parts of the ossicles such as the annular ligament, which is difficult to evaluate intraoperatively by palpation. Such partial fixation may be present preoperatively or develop later on. This clarification is uncertain because of the non-availability of a sensitive technique for measurement of the mobility of ossicles intraoperatively (25).

Ascertaining the reason for failure in the improvement of hearing and their prevention in future surgeries is of utmost importance. In our study, the failure group comprised of six cases, four of which had clinical signs of eustachian tube dysfunction. Two of these patients had good graft uptake but the post-operative hearing was subnormal. Revision surgery was done in one of these cases. Intraoperatively glass ionomer cement was found broken into pieces. It was removed by suctioning and Rebridging ossiculoplasty was performed again.

In the remaining one unsuccessful case, the patient was counseled for revision surgery keeping in mind the fixation of stapes suprastructure. The patient declined revision surgery. The neotympanum was normal, middle ear well ventilated, but ABG on PTA was in the range of 21-27dB.

There are some important points to be kept in mind during the glass ionomer cement application. Any bleeding in the middle ear should be controlled beforehand, and the application should be done within two minutes of mixing when it has the most suitable consistency. Mucosa covering ossicles should be detached and then a drop of GIC should be applied as bone cement doesn’t stick to the mucosa (26,27). Care should be taken to prevent bone cement from coming in direct contact with perilymph, dura mater, or any of the neural structures because of its possible neurotoxic side effects. (12,27,28).

In case of any inadvertent contamination, the cement should be suctioned immediately, and the area irrigated with saline. Also, a small piece of gel foam can be used over critical structures such as facial nerve and stapes footplate for their protection (18). 

Nevertheless, some adverse effects of ionomeric bone cement were reported including encephalopathy, gliosis, and facial nerve paralysis (29,30). But this was mostly encountered when GIC was used in large volumes such as in mastoid obliteration and posterior ear canal wall reconstruction. 

A systematic review done by Wegner et al. showed that seven studies had no infection or extrusion reported in patients in which ossiculoplasty with GIC was performed (25,31–35). Celenk et al. study showed a single case of granulation over the ossicles (2%) and one case of extrusion (2%) (16). A study by Rath et al. showed one case of disintegrated GIC requiring revision surgery(3%) (25).

We did not find any such complication in our 25 patients except in one case in which the failure of graft uptake and no improvement of hearing prompted us to do revision surgery. During revision surgery, GIC was found broken into pieces in the middle ear. Apart from this, no major side effects of GIC use were observed in ossicular reconstruction.

Long term results of GIC ossiculoplasty is still a topic for debate. We conclude that the GIC was simple to use and saves considerable operative time and money especially in developing countries, where expensive treatment and prostheses are not affordable for the majority of the patients.

## Conclusion

GIC has been used in modern times with good results. In our study, we observed that GIC was simple to use and saves considerable operative time and money. It also imparted good hearing results and indicate more trials should be conducted using this method.

## Limitations

There is a lack of literature determining the supremacy of Glass Ionomer Cement over other ossiculoplasty techniques, due to variations in sample size characteristics and geography, different socioeconomic conditions of previous studies, and varying patient characteristics. We recommend a similar study with a larger and more diverse sample and a longer follow‑up period to analyze the results of using GIC as an ossiculoplasty material.
